# New Half-Metallic Materials: FeRuCrP and FeRhCrP Quaternary Heusler Compounds

**DOI:** 10.3390/ma10121367

**Published:** 2017-11-28

**Authors:** Jiannan Ma, Liefeng Feng, Ruikang Guo, Yi Liao, Rabah Khenata, Guodong Liu, Liying Wang

**Affiliations:** 1 Tianjin Key Laboratory of Low Dimensional Materials Physics and Preparing Technology, Faculty of Science, Tianjin University, Tianjin 300350, China; majiannan@tju.edu.cn (J.M.); fenglf@tju.edu.cn (L.F.); yi.liao@tju.edu.cn (Y.L.); 2 School of Materials Sciences and Engineering, Hebei University of Technology, Tianjin 300130, China; guork1993@126.com (R.G.); gdliu1978@126.com (G.L.); 3 Laboratory of Quantum Physics and Mathematical Modeling (LPQ3M), Department of Technology, University of Mascara, 29000 Mascara, Algeria; khenata_rabah@yahoo.fr

**Keywords:** first-principles calculations, half-metallic properties, quaternary Heusler compound

## Abstract

The electronic structures and magnetic properties of FeRuCrP and FeRhCrP quaternary Heusler compounds with LiMgPbSb-type structures have been investigated via first-principles calculations. The calculational results show that both FeRuCrP and FeRhCrP compounds present perfect half-metallic properties: Showing large half-metallic band gaps of 0.39 eV and 0.38 eV, respectively. The total magnetic moments of FeRuCrP and FeRhCrP are 3 μB and 4 μB per formula unit, respectively. The magnetism of them mainly comes from the 3d electrons of Cr atoms and follows the Slater-Paulig behavior of Heusler compounds: *M_t_* = *Z_t_* − 24. Furthermore, the half-metallic properties of FeRuCrP and FeRhCrP compounds can be kept in a quite large range of lattice constants (about 5.44–5.82 Å and 5.26–5.86 Å, respectively) and are quite robust against tetragonal deformation (c/a ratio in the range of 0.94–1.1 and 0.97–1.1, respectively). Moreover, the large negative cohesion energy and formation energy of FeRuCrP and FeRhCrP compounds indicate that they can be synthesized experimentally.

## 1. Introduction

The half-metallic (HM) ferromagnets, which exhibit metallic property in one spin channel, while the other spin band shows semiconducting characteristics, have received a lot of attention since they were predicted in half-Heusler compound NiMnSb (C1_b_ structure and space group $F\bar{4}3m$) by de Groot et al. in 1983 [1]. Due to the unique band structure character, HM ferromagnets show a complete (100%) conduction electron spin polarization. Thus, a HM ferromagnet would be a promising candidate for a spintronic device. Many HM ferromagnets with different structures have been predicted to exhibit half-metallicity [2,3,4,5,6,7,8,9,10,11,12,13,14,15,16,17,18,19,20]. Among them, Heusler compounds (ternary intermetallic compounds with the general formula X_2_YZ) are a large family in which to find new HM ferromagnets, because they usually have high Curie temperature and are easily synthesized experimentally. 

Recently, one of the family of Heusler compounds—quaternary Heusler compounds with chemical formula of XMYZ (all X, M, Y, Z are different atoms and the structural prototype of the quaternary compound is LiMgPdSb, space group of $F\bar{4}3m$)—has attracted much attention. More and more quaternary Heusler compounds are found to show HM properties [21,22,23,24,25,26,27]. Meanwhile, most researchers mainly investigate the HM properties of the quaternary Heusler compounds that contain the 3d transition-metal atoms, such as CoFeCrZ (Z = Al, Si, Ga and Ge) [21], CoFeMnZ (Z = Al, Ga, Si and Ge) [22,23,24] and NiFeTiZ (Z = Si, P, Ge and As) [25]. 

More recently, the quaternary Heusler compounds containing 4d elements began to attract researchers’ attention. In addition, some new half-metals have been found in this field, such as CoRhMnZ (Z = Ge, Si) [28], CoRuTiZ (Z = Si, Ge and Sn) [29], TiZrCoIn [30], ZrVTiZ (Z = Al, Ga) [31], ZrCoTiZ (Z = Si, Ge, Ga and Al) [32] and ZrMnVZ (Z = Si, Ge) [33]. It was reported that the HM band gap of the quaternary Heusler compounds with 4d transition elements is usually larger than that of the compounds only containing 3d transition elements [34]. In addition, this will be conducive to the stable synthesis of the half-metallic materials in practical application.

As we all know, many Fe_2_-based Heusler compounds have been proved to be half-metal in theory and experimentally [35]. Among them, Fe_2_CrP Heusler compound is one of the typical half-metals with an energy band gap of 0.22 eV [36]. In this paper, we substitute one of the Fe atoms with Ru or Rh atoms, forming two new quaternary Heusler compounds: FeRuCrP and FeRhCrP. We have investigated the electronic structures and magnetic properties of them by using first-principles calculations [37]. Moreover, the half-metallicity and magnetism of these compounds under hydrostatic strain and tetragonal deformation have also been discussed in detail.

## 2. Calculation Details

The electronic structure and magnetism of LiMgPbSb-type FeRuCrP and FeRhCrP compounds were calculated by using the pseudopotential plane-wave method [38] as implemented in the Cambridge Serial Total Energy Package (CASTEP) code [39]. The CASTEP code is an effective ab initio program based on quantum mechanics. It can precisely simulate the ground structure, band structure, optical properties, magnetic properties, and so on. In the CASTEP code, the pseudopotential method with a plane-wave basis set is used. The interactions between the atomic core and the valence electrons were described by the ultrasoft pseudo-potential approach. The exchange correlation within the generalized gradient approximation (GGA) based on Perdew-Burke-Ernzerhof (PBE) was performed for calculation [40,41,42]. The cut-off energy of the density plane-wave was set as 400 eV. A mesh of 12 × 12 × 12 k-points in the full irreducible Brillouin Zone was used for all of the cases. The convergence tolerance was chosen as a difference in total energy within $5\times 10^{-7}$ eV per atom.

## 3. Results and Discussion

**Figure 1:**
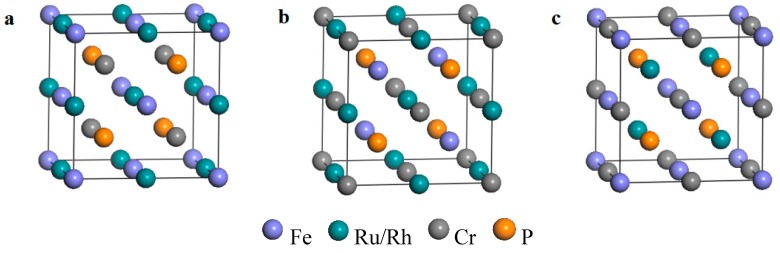
Schematic illustration of the three possible nonequivalent structures of FeRuCrP and FeRhCrP quaternary Heusler compounds (**a**) type 1, (**b**) type 2 and (**c**) type 3.

**Figure 2:**
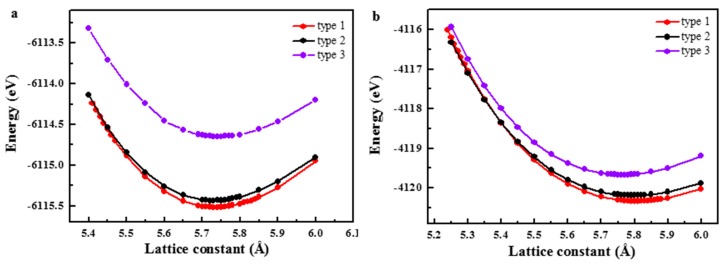
The calculated total energy as a function of the lattice constant for (**a**) FeRuCrP (**b**) FeRhCrP quaternary Heusler compounds with three different structures.

For the LiMgPbSb-type quaternary Heusler compounds Fe-Ru-Cr-P and Fe-Rh-Cr-P, there are three possible different atom arrangement (as shown in Table 1 and Figure 1). In the Wyckoff coordinates, type 1 (FeRu/RhCrP): Fe atoms occupy 4a (0, 0, 0), Ru/Rh atoms occupy 4c (1/2, 1/2, 1/2), Cr atoms occupy 4b (1/4, 1/4, 1/4) and P atoms occupy 4d (3/4, 3/4, 3/4); type 2 (CrRu/RhFeP): Cr 4a (0, 0, 0), Ru/Rh 4c (1/2, 1/2, 1/2), Fe 4b (1/4, 1/4, 1/4) and P 4d (3/4, 3/4, 3/4); type 3 (FeCrRu/RhP): Fe 4a (0, 0, 0), Cr 4c (1/2, 1/2, 1/2), Ru/Rh 4b (1/4, 1/4, 1/4) and P 4d (3/4, 3/4, 3/4). Firstly, the total energy as a function of lattice constants (E_t_/a curves) of FeRuCrP and FeRhCrP compounds with the three different atomic arrangement were calculated to confirm the ground state structure of them, the optimized results are shown in Figure 2. From Figure 2, one can see that for both the FeRuCrP and FeRhCrP compounds, due to the lowest total energy, the structure of type 1 (FeRh/RuCrP) is the ground state structure among the three different configurations, which indicates that FeRuCrP and FeRhCrP compounds prefer crystallizing in the type 1 structure. Next, we will discuss the electronic structure and magnetic properties of the FeRuCrP and FeRhCrP compounds only for the most stable type 1 structure.

**Figure 3:**
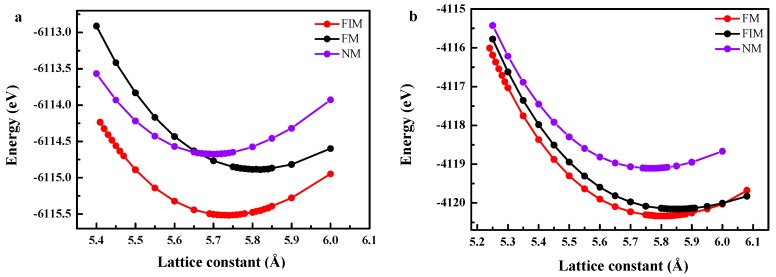
The calculated total energy as a function of the lattice constant for (**a**) FeRuCrP (**b**) FeRhCrP quaternary Heusler compounds. FIM, FM and NM correspond to ferromagnetic, ferromagnetic and nonmagnetic calculations.

In order to confirm the magnetic ground state of FeRuCrP and FeRhCrP compounds with type 1 structure, the total energies of the non-magnetic (NM), ferrimagnetic (FIM) and ferromagnetic (FM) states as a function of the lattice constants of the FeRuCrP and FeRhCrP compounds were calculated (as shown in Figure 3). The results show that the FeRuCrP and FeRhCrP compounds present different magnetic ground states. For FeRuCrP compound, it can be seen that FIM state is the ground state, while the FM state is the ground state for FeRhCrP compound. It is worth noting that, for FeRhCrP compound, with the lattice constant expansion, a FIM-FM intersection was observed, which indicates that the material can exhibit a FIM-FM transition under a proper external driving force (i.e., stress). Therefore, it can be interesting to investigate the competition of the FIM and FM states of FeRhCrP quaternary Heusler compound in further studies.

The obtained equilibrium lattice parameters of FeRuCrP and FeRhCrP compounds are 5.74 Å and 5.80 Å, respectively. Obviously, the quite similar equilibrium lattice parameters of FeRuCrP and FeRhCrP compounds are due to the similar atomic radius of Ru and Rh atoms, which are adjacent in the Periodic Table. 

The total and atomic magnetic moments of FeRuCrP and FeRhCrP compounds with the equilibrium lattice parameters were gathered in Table 2. From Table 2, it can be seen that the calculated total magnetic moments per formula unit are 3 *μ_B_* and 4 *μ_B_* for FeRuCrP and FeRhCrP compounds, respectively, which mainly derive from the Cr atoms. According to the theories of the origin of the energy gap in the Ferromagnetic Heusler alloys [43], the number of the valence electrons of FeRuCrP and FeRhCrP are 27 and 28, then, the total magnetic moments are 3 *μ_B_* and 4 *μ_B_*, respectively. Our calculation results are consistent with Galanakis’s study. One can state that the total magnetic moments of these compounds are integral values, obeying the Slater-Pauling rule of half-metallic ferromagnets with Heusler structure [43]:

(1) $$M_{t}=Z_{t}-24$$

where *M_t_* and *Z_t_* represent the total magnetic moment per formula unit and the number of the total valence electrons accumulated in the systems. For FeRuCrP compound, the Fe and Cr atoms have negative magnetic moments compared to the Ru atom and the calculated atomic magnetic moments are Fe (0.36 *μ_B_*), Ru (−0.40 *μ_B_*), Cr (3.04 *μ_B_*) and P (0.02 *μ_B_*), respectively, which is consistent with the FIM state discussed above. Meanwhile, the FeRhCrP compound, due to the parallel atomic magnetic arrangements of Fe (0.60 *μ_B_*), Rh (0.26 *μ_B_*), Cr (3.12 *μ_B_*) and P (0.04 *μ_B_*) atoms, exhibits a ferromagnetic character. 

**Figure 4:**
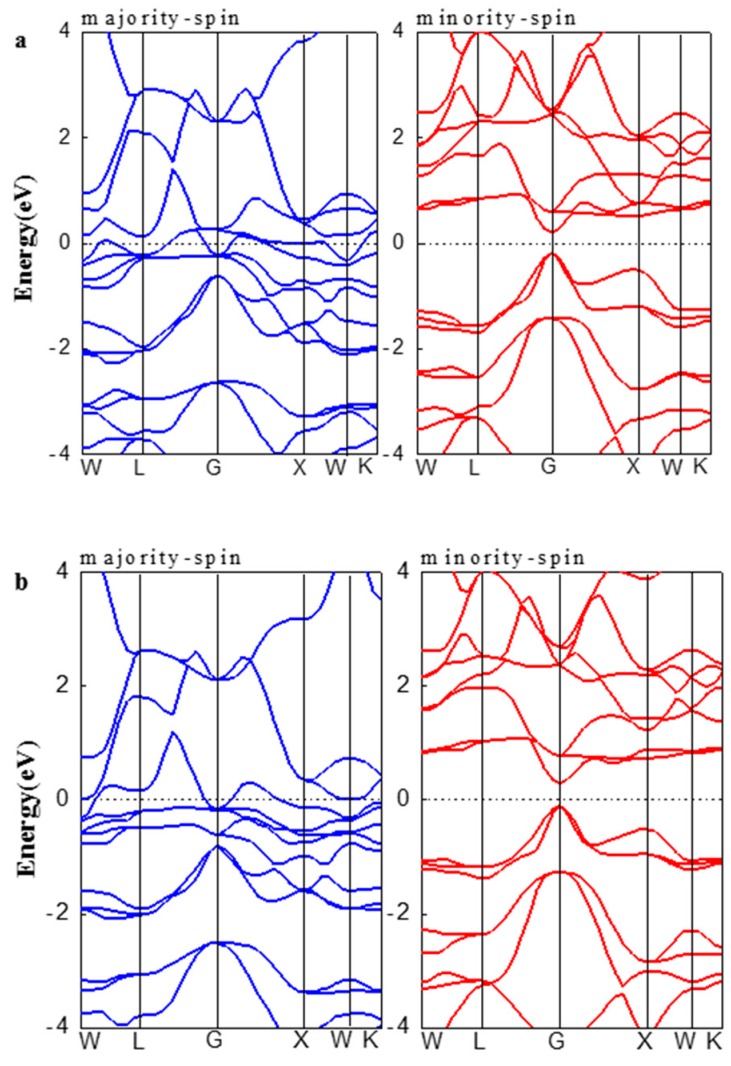
The band structure of (**a**) FeRuCrP and (**b**) FeRhCrP quaternary Heusler compounds at the equilibrium lattice constants. The dashed line indicates the Fermi level at 0 eV.

The calculated spin-polarized band structures of FeRuCrP and FeRhCrP quaternary Heusler compounds at their equilibrium lattice constants are displayed in Figure 4a,b. It can be clearly seen that the band structures around the Fermi level of FeRuCrP and FeRhCrP compounds are quite similar. The majority-spin bands show a metallic property, while a direct energy band gap near Fermi level is opened in the minority-spin channel. Moreover, FeRuCrP and FeRhCrP quaternary Heusler compounds have almost the same robust energy gaps: 0.39 eV and 0.38 eV, respectively. This result is a 100% spin polarization of the conduction electrons and makes FeRuCrP and FeRhCrP quaternary Heusler compounds half-metals.

**Figure 5:**
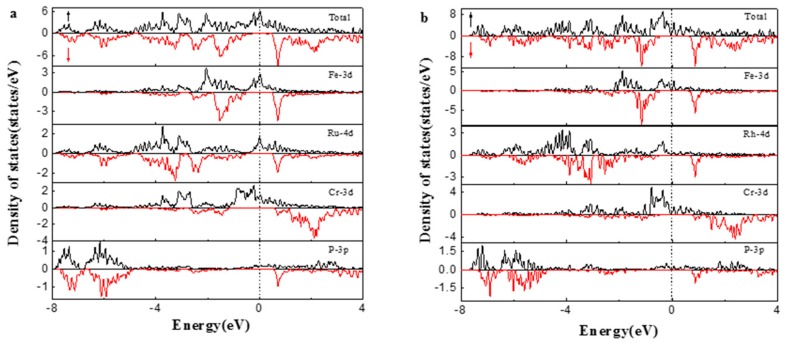
The total and partial density of states (DOS) of (**a**) FeRuCrP (**b**) FeRhCrP quaternary Heusler compounds at the equilibrium lattice constants. The dashed line indicates the Fermi level at 0 eV.

In order to understand the electronic structure of FeRuCrP and FeRhCrP quaternary Heusler compounds further, the density of states (DOS) of them are presented in Figure 5. From Figure 5, it can be seen that for both the FeRuCrP and FeRhCrP quaternary Heusler compounds, there is a metallic intersection with the Fermi level in the majority-spin channel, while in the minority-spin bands, the Fermi level just locates in the energy band gap. Therefore, the FeRuCrP and FeRhCrP quaternary Heusler compounds are half-metals, which is consistent with the above discussion on the band structures quite well. As we all know, in alloys, valence electron energy bands are all hybridized states which arise from the hybridization of the electronic states between atoms. From the partial density of states (PDOS), it can be seen that, in some energy regions, the shape of the PDOS distribution of the neighboring atoms are very similar. These energy regions are usually identified as the hybrid regions. The bonding states in the low-energy region and antibonding states in the high-energy region can be formed in the process of the electron hybridization between atoms. For FeRuCrP compound, in the energy region from −5 eV to 1 eV, one can see that the minority DOS peaks below E_f_ mainly originate from the bonding states of Fe 3d (−3 eV to 1 eV) and Ru/Rh 4d (−5 eV to 1 eV/−4.5 eV to 1 eV), while above E_f_ mainly come from the hybridization of Fe 3d and Ru/Rh 4d states. In addition, in the majority DOS, the total DOS shows a three-peak structure, which mainly comes from the hybridization of the d states of Fe and Ru/Rh and bonding states of Cr 3d states. In the energy region from 1 eV to 4 eV, it is the contribution of the antibonding states of Cr. Moreover, it should be noted that Cr atom has a strong spin-splitting both for FeRuCrP and FeRhCrP compounds. Therefore, Cr atoms are the main contributors to the total magnetic moment. Since Rh atom has one more valence electron than Ru, the majority DOS of FeRhCrP is shifted to a lower energy region compared to FeRuCrP. 

It is known that not all the theoretical predicted material can be synthesized and form a stable phase experimentally. To test that the FeRuCrP and FeRhCrP quaternary Heusler compounds can be synthesized and stabilized experimentally, the cohesion energy *E_coh_* and formation energy *E_for_* were calculated. The relevant results were presented in Table 2.

The cohesion energy (*E_coh_*) was calculated by:

(2) $$E_{coh}=E_{tot}-E_{Fe}^{\mathrm{iso}}-E_{Ru}^{iso}-E_{Cr}^{iso}-E_{P}^{iso}$$

where $E_{tot}$ is the equilibrium total energy calculated from first-principles of the FeRuCrP and FeRhCrP quaternary Heusler compounds per formula unit, and $E_{Fe}^{iso}$, $E_{Ru}^{iso}$, $E_{Cr}^{iso}$ and $E_{P}^{iso}$ correspond to the energies of each isolated atom, respectively. The calculated values of the cohesion energy are −19.95 eV for FeRuCrP and −17.27 eV for FeRhCrP. Such high and negative cohesion energy indicates the high energy of the chemical bonds. Therefore, the quaternary Heusler compounds FeRuCrP and FeRhCrP are expected to form a stable phase experimentally. 

The formation energy is calculated using the formula:

(3) $$E_{for}=E_{tot}-E_{Fe}^{bulk}-E_{Ru}^{bulk}-E_{Cr}^{bulk}-E_{P}^{bulk}$$

where $E_{F_{e}}^{bulk}$, $E_{R_{u}}^{bulk}$, $E_{C_{r}}^{bulk}$ and $E_{P}^{bulk}$ are the total energy per atom in the bulk. The calculated formation energies of FeRuCrP and FeRhCrP are −2.82 eV and −3.30 eV, which indicate that the FeRuCrP and FeRhCrP quaternary Heusler compounds are thermodynamically stable. Thus, FeRuCrP and FeRhCrP quaternary Heusler compounds are possible to be synthesized and stabilized experimentally. 

**Figure 6:**
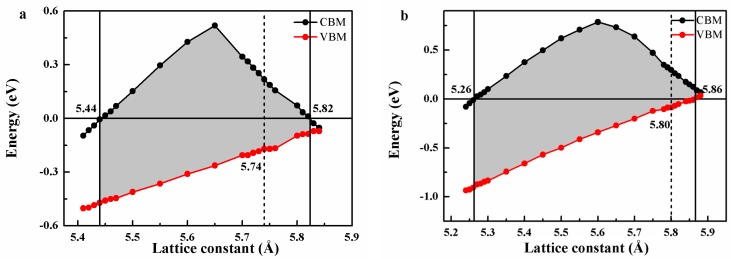
The minority-spin band gaps of (**a**) FeRuCrP (**b**) FeRhCrP quaternary Heusler compounds under the hydrostatic strain. The red and black squares show the valence band minimum (VBM) and conduction band maximum (CBM), respectively. The horizontal dashed line indicates the Fermi level at 0 eV.

Note that the lattice thermal expansion and the external stress often result in the destruction of the half-metallicity. In order to examine the effect of thermal expansion and external stress on the half-metallicity, the band structures of FeRuCrP and FeRhCrP quaternary Heusler compounds at different lattice constants were calculated. The values of the valence band maximum (VBM) and conduction band minimum (CBM) in the minority-spin direction are used to describe the change of the half-metallicity under the different lattice deformation for FeRuCrP and FeRhCrP compounds (as shown in Figure 6). From Figure 6, it can be found that the half-metallicity can be retained in a quite large constant range of 5.44–5.82 Å and 5.26–5.86 Å for FeRuCrP and FeRhCrP compounds, respectively. Meanwhile, the Fermi level has an intersection with the conduction bands when the lattice constants are smaller than 5.44 Å and 5.26 Å for FeRuCrP and FeRhCrP compounds, respectively. Thus, the half-metallicity of these compounds was lost although the band gap is still retained in the minority-spin channel. On the other hand, when the lattice constant is expanded to 5.82 Å and 5.86 Å for FeRuCrP and FeRhCrP compounds, different from the cases of the lattice compression, the half-metallicity was lost due to the overlapping of the Fermi level with the valence and conduction bands. In other words, FeRuCrP and FeRhCrP compounds can maintain the half-metallicity in the stress range of –5.23–1.39% and −9.31–1.03%. Thus, it can be said that the half-metallicity of the FeRuCrP and FeRhCrP compounds is quite robust against the lattice change, which indicates that these two compounds would be good candidates for spintronic device that applying in a quite wider temperature range.

**Figure 7:**
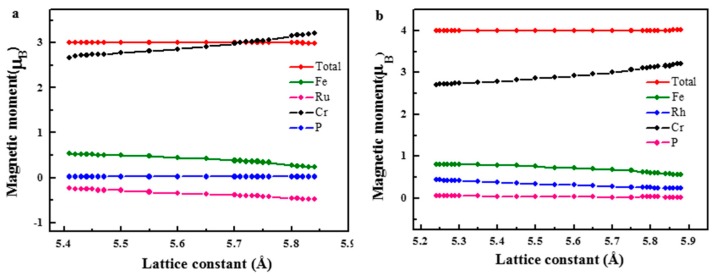
The total and partial magnetic moments as a function of lattice constant for (**a**) FeRuCrP (**b**) FeRhCrP quaternary Heusler compounds.

In addition, the magnetic moment (including total and atomic magnetic moment) as a function of the lattice constant for the FeRuCrP and FeRhCrP compounds are shown in Figure 7. From Figure 7, one can see that the values of the total magnetic moments for FeRuCrP and FeRhCrP compounds are still 3 μB and 4 μB in the range of 5.44–5.82 Å and 5.26–5.86 Å, respectively. Furthermore, the total magnetic moments of them still follow the *M_t_* = *Z_t_* − 24 rule within the corresponding lattice constant range. For Fe and Cr atoms, their atomic magnetic moments are sensitive to the change of lattice constant in the range of 5.2–6.0 Å (FeRuCrP) and 5.0–6.0 Å (FeRhCrP). Meanwhile, for the Ru/Rh and P atoms, their magnetic moments almost retain constants with the change of the lattice constant in the wide lattice constant range. With the lattice expansion, the atomic magnetic moments of Fe and Cr atoms show a gradually increasing trend. Due to the antiparallel arrangement of Fe and Cr atoms, the increasing magnetic moment offsets each other and the total magnetic moment keeps a constant.

**Figure 8:**
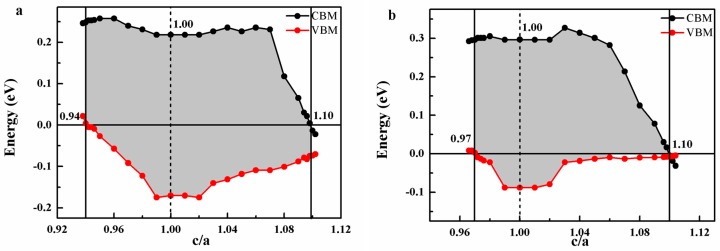
The minority-spin band gap of (**a**) FeRuCrP (**b**) FeRhCrP quaternary Heusler compounds under the tetragonal deformation. The red and black squares show the valence band minimum (VBM) and conduction band maximum (CBM), respectively. The horizontal dashed line indicates the Fermi level at 0 eV.

In practical application, tetragonal distortion is an important factor to damage the half-metallicity when the compounds are fabricated into film. In order to simulate the influence of tetragonal distortion on the half-metallicity, the CBM and VBM values under different c/a ratio were also calculated (shown in Figure 8). It can be seen that FeRuCrP compound can maintain the half-metallic property when c/a ratio is in the range of 0.94–1.10. The largest gap is 0.39 eV at c/a = 1.0 and the width of the gap decreases when c/a ratio deviates from c/a = 1.0 ratio to be a smaller of bigger value. For FeRhCrP compound, the similar characteristics can be observed and the half-metallic characteristic can be held when c/a is in the range of 0.97–1.10 and the widest energy gap also locates at the c/a = 1.0 ratio.

**Figure 9:**
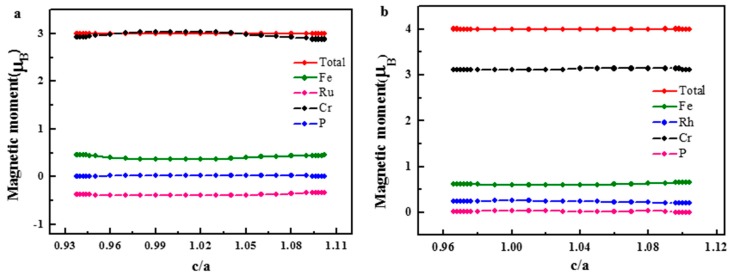
The total and partial magnetic moments as a function of c/a ratio for (**a**) FeRuCrP (**b**) FeRhCrP quaternary Heusler compounds.

The total and atomic magnetic moments as a function of the c/a ratio are shown in Figure 9. It can be seen that both the total and atomic magnetic moments of the FeRuCrP and FeRhCrP compounds almost keep unchanged throughout the whole tetragonal deformation (c/a = 0.9–1.1). When the c/a is larger or smaller than the critical values, the FeRuCrP and FeRhCrP compounds become the ordinary ferri- and ferromagnets.

We have also investigated the effect of the lattice distortion on the majority-spin bands for FeRuCrP and FERhCrP compounds. The calculational results show that, with the changing of the lattice parameter and the c/a ratio, there was no obvious change of the spin splitting. Thus, the Fermi level always locates at a DOS peak in the majority-spin channel for FeRuCrP and FERhCrP compounds. While, as discussed above, the Fermi level locate at an energy gap in the minority-spin channel and the energy gap can be kept in a quite large range of lattice constants and c/a ratio. Therefore, the half-metallicity of FeRuCrP and FeRhCrP compounds would be extremely stable against the external strain and stress.

## 4. Conclusions

In summary, the robust half-metallicity has been predicted in FeRuCrP and FeRhCrP quaternary Heusler compounds by first-principles calculations. The FeRuCrP and FeRhCrP quaternary Heusler compounds are half-metallic ferri- and ferromagnets, respectively, with the total magnetic moments of 3 μB and 4 μB per unit cell, respectively. The magnetism of them mainly originates from the 3d electrons of Cr atoms and follows the Slater-Paulig behavior: *M_t_* = *Z_t_* − 24. Their half-metallicity is robust to the lattice compression and tetragonal distortion. It was known that half-metals are excellent providers of high-spin polarized current. For example, they can used as the source material for spin injection and the electrode materials for giant magnetoresistance (GMR) devices, etc. Therefore, the FeRuCrP and FeRhCrP quaternary Heusler compounds with robust half-metallicity are promising materials for future spintronic devices.

## Figures and Tables

**Table 1:** Three possible different types of atom arrangement of FeRuCrP and FeRhCrP quaternary Heusler compounds.

Structure	Fe	Ru/Rh	Cr	P
type 1	4a	4c	4b	4d
type 2	4b	4c	4a	4d
type 3	4a	4b	4c	4d

**Table 2:** The calculated equilibrium lattice constant *a*_0_ (Å), total and partial magnetic moments (*μ_B_*) at the equilibrium lattice constant, the formation energy (*E_for_*/eV) and cohesive energy (*E_coh_*/eV) both for FeRuCrP and FeRhCrP quaternary Heusler compounds.

Compounds	*a*_0_	*M_tot_*	*M_Fe_*	*M_Ru_/M_Rh_*	*M_Cr_*	*M_P_*	*E_for_*	*E_coh_*
FeRuCrP	5.74	3.00	0.36	−0.40	3.04	0.02	−2.82	−19.95
FeRhCrP	5.80	4.00	0.60	0.26	3.12	0.04	−3.30	−17.27
